# Knowledge of non-communicable diseases (NCDs) is less strongly associated with self-management of NCDs and quality of life in older age in Phayao, Thailand: A moderated mediation analysis

**DOI:** 10.1371/journal.pone.0362216

**Published:** 2026-11-11

**Authors:** Nestor Asiamah, Manuchet Manotham, Wannita Sakulwattana, Parichat Ong-Artborirak, Supakan Kantow, Prapaporn Tulwattanakul, Esther Liyanage, Supandee Maneelok, Katekaew Seangpraw

**Affiliations:** 1 School of Health and Social Care, University of Essex, Colchester, United Kingdom; 2 School of Public Health, University of Phayao, Phayao, Thailand; 3 Department of Research and Medical Innovation, Faculty of Medicine Vajira Hospital, Navamindradhiraj University, Bangkok, Thailand; 4 Department of Physiotherapy, Faculty of Allied Health Sciences, University of Peradeniya, Kandy, Sri Lanka; 5 Faculty of Health and Sports Science, Thaksin University Phatthalung Campus, Phatthalung, Thailand; Instituto Nacional de Ciencias Medicas y Nutricion Salvador Zubiran, MEXICO

## Abstract

Non-communicable diseases (NCDs) are a major global health concern and are associated with lower health-related quality of life (HRQoL). The objective of this study was to investigate whether the indirect association of knowledge of NCDs with HRQoL through self-management of NCDs is moderated by age among older adults living in communities in Phayao Province, Thailand. A cross-sectional design was employed, and 424 older adults selected with the convenience sampling method participated in the study. Key variables included and quantified with standard scales were knowledge, self-management, and quality of life (WHOQOL-OLD). Data were analysed with Hayes’ PROCESS Macro through a moderated mediation model. Knowledge was positively associated with HRQoL and self-management of NCDs but associated with age. HRQoL was positively associated with self-management of NCDs, and self-management of NCDs partially mediated the positive association of knowledge and HRQoL. The association between knowledge and self-management of NCDs was weaker in older age groups. A negative moderated mediation was confirmed, suggesting that the positive indirect association of knowledge with HRQoL through self-management of NCDs was weaker in older age. Knowledge was associated with better self-management of NCDs and HRQoL among older adults. The association of knowledge with HRQoL was weaker in older age. Knowledge-based interventions aimed at improving self-management of NCDs in older age can enhance HRQoL.

## Introduction

Non-communicable diseases (NCDs) remain a significant public health challenge and are the primary cause of illness and death, accounting for up to 43.0 million deaths annually—approximately 75.0% of all deaths worldwide [1]. The five leading NCDs contributing to mortality are cancer, cardiovascular diseases, diabetes, hypertension, and stroke [1,2]. Furthermore, individuals with NCDs often experience multimorbidity, defined as the coexistence of two or more chronic conditions in the same individual [1]. The prevalence of multimorbidity in the general population worldwide ranges from 13.0% to 72.0%, depending on age group [3]. This situation reflects the continuously increasing burden of disease, primarily driven by demographic structural changes, particularly the rapid growth in the number and proportion of older adults, which is a trend observed in Thailand and globally [1,4].

Thailand has entered the stage of a super-aged society, with more than 14.37 million people aged 60 years or higher, accounting for approximately 21.8% of the total population [5]. Over 38.0% of older adults have at least two chronic diseases, including hypertension, diabetes, and heart disease [2,5]. In addition, older adults account for more than 68.0 million outpatient visits annually, reflecting the growing burden on Thailand’s public health system [5]. Based on regional analysis, Health Service Region 1 in Northern Thailand (comprising Chiang Mai, Chiang Rai, Mae Hong Son, Phayao, Phrae, and Nan) has experienced an increase of more than 20.0% in the number of patients with NCDs [2]. In Thailand, rapid economic growth combined with systemic challenges in healthcare services and a lack of integrated proactive health promotion, particularly for older adults, has increased the risk of NCDs [2]. National efforts have focused on various interventions, yet they have not curbed rising NCD prevalence, which adversely impacts quality of life (QoL) [5]. Several studies indicate that older adults with NCDs lack adequate awareness of their chronic conditions and insufficient self-management skills, and this negatively affects quality of life [6–8]. The presence of NCDs in older adults significantly impacts health-related quality of life (HRQoL) and independence [8,9]. Older adults living with NCDs often exhibit low HRQoL [8–10]. Moreover, studies in Thailand have found a negative association between age and both physical and mental HRQoL, with the latter highlighting the vulnerability of older adults to mental health decline [8]. This situation calls for interventions aimed at improving self-management of NCDs among older adults in Thailand.

Self-management is a process whereby individuals initiate and carry out independent activities to sustain life, health, and overall well-being. It encompasses physical, psychological, and behavioral aspects of daily living [11,12]. The World Health Organization (WHO) has noted that chronic disease management should focus on empowering individuals and communities to effectively promote patients’ self-management behaviors rather than relying solely on other interventions [13]. A systematic review has identified that self-management of chronic NCDs can facilitate rehabilitation, control disease progression, and prevent complications [14]. However, patients with chronic diseases often exhibit poor self-management skills, which may be associated with low self-efficacy [6]. Deficient self-confidence has been identified as a factor that compromises self-management in chronic disease control, thereby contributing to a deterioration in QoL [6,15]. Therefore, understanding the association between NCD knowledge and both HRQoL and the self-management of NCDs is essential for future interventions [16]. In Thailand, there is a paucity of studies assessing the foregoing nexus, and no studies have focused on individuals living with NCDs. Previous research has focused solely on diabetic patients.

The WHO (1995) defines QoL as an individual’s perception of their position in life within the context of the culture and value systems in which they live, in relation to their goals, expectations, standards, and personal concerns [17]. This reflects a comprehensive concept that encompasses the physical, psychological, social, and environmental dimensions that influence an individual’s life [17]. Previous literature reviews have indicated that QoL may vary across different age groups and health conditions [18], which calls for a study focused on older adults with NCDs [8,18]. HRQoL focuses on the individual’s well-being and, hence, reflects general health. This study therefore employs the WHOQoL-Old instrument, which was specifically designed to assess HRQoL in older adults [8,17,18]. The WHOQoL-Old comprises six factors: (1) sensory abilities; (2) autonomy; (3) past, present, and future activities; (4) social participation; (5) death and dying; and (6) intimacy [18]. The WHOQoL-Old is a suitable instrument for assessing HRQoL because of its multidimensional structure [18, 19].

National interventions in Thailand have been implemented at both primary and secondary care levels to strengthen knowledge related to NCDs and to promote patient self-management, including health education programs, screening services, and chronic disease clinics [5]. Despite these efforts, the incidence of NCDs continues to increase, and the QoL among individuals living with NCDs varies across age groups, with older adults consistently reporting lower HRQoL [7,8,18]. Although ageing may provide opportunities for experiential learning and skill development, older adults in rural areas often face structural barriers such as poverty, limited educational attainment, and weaker social networks, which may restrict their access to and understanding of NCD-related information [20,21]. Consequently, older age in rural settings may be associated with lower levels of NCD knowledge. In such contexts, age may influence how NCD knowledge relates to both self-management behaviors and QoL among rural residents. This perspective suggests a moderated mediation mechanism in which age modifies the indirect relationship between NCD knowledge and QoL through self-management of NCDs. To date, this potential moderated mediation has not been empirically examined in the literature despite its important implications for public health practice and intervention development in ageing rural populations.

The above moderated mediation has two parts: (1) the mediation of the link between knowledge of NCDs and QoL by self-management of NCDs, and (2) the moderating role of age in the relationship between knowledge and self-management of NCDs. Both the moderating and mediation roles tested are statistical (structural) but not causal, given that data from a cross-sectional study were used in this study. Our assessment of this relationship is expected to improve stakeholders’ understanding of how the variables relate and provide information for generating or refining hypotheses and assessing causal mechanisms in future experimental research. Implications for practice and future research are delineated.

## Methods and materials

### Design and study setting

This study utilized a cross-sectional design, with data collected between November 2024 and April 2025 in the Pong District, a remote highland region in Northern Thailand, situated far from the city of Phayao.

### Selection and ethical considerations

The study was conducted in rural communities in Pong District, Phayao Province, where the number of older adults with non-communicable diseases (NCDs) had increased by approximately 5%. First, one subdistrict, Pha Chang Noi, was randomly selected from the seven subdistricts in Pong District using a lottery method. Pha Chang Noi consists of eight villages. Because eligible older adults with NCDs were distributed across these villages and a complete operational sampling frame was not available for probability-based recruitment, convenience sampling was used to recruit accessible and willing village residents who met the predefined eligibility criteria. Recruitment continued until the required sample size was reached.

The study’s sample size was determined using Khazaie’s formula [22], considering the large and uncertain population in the research area. With a confidence level of 95% and a 5% margin of error, the calculated sample size was 385 individuals. To account for potential missing data, an additional 10% (n = 39) was added to the calculated sample, bringing the final sample size to 424. Following approval from the human research ethics committee at the University of Phayao (HREC-UP-HSST 1.3/018/67), we collaborated with community leaders, village health volunteers, village heads, and public health officers at the local primary care unit to broadcast project details via loudspeakers, encouraging residents to volunteer for the study. Participants were selected based on the following criteria: an age of 60 years or older, a diagnosis from a physician of one or more NCDs of at least six months duration, residence in the study area for no less than 1 year, an ability to communicate in the local language, and an agreement to participate by signing a written consent form. The study excluded participants who had cognitive or mental health disorders.

### Data collection

Before the research commenced, the subdistrict initiated the recruitment of 10 research assistants, including three public health experts and seven village health volunteers. They possessed local language skills, cultural knowledge, and the ability to reach the sample group. Before collecting data, local research assistants attended a 2-hour briefing to discuss the goals of data collection, learn proper techniques for handling questionnaires, and review fundamental study information. Interview times were scheduled, and ethical procedures were followed. The research team and assistants gathered data at the Ban Pang Ka Subdistrict Health Promotion Hospital and the community’s multipurpose building between 08:30 and 12:00 hrs. Each participant was interviewed and was asked to complete a questionnaire, which lasted 10–15 minutes.

### Ethical considerations

This study was approved by the University of Phayao Research Ethics Committee on Human Research (HREC-UP-HSST 1.3/018/67). Informed consent was obtained from all participants prior to their inclusion in the study. Participants were provided with a detailed consent form that outlined the purpose of the research, the procedures involved, potential risks and benefits, and their right to withdraw from the study at any time without any consequences. All procedures performed in studies were in accordance with the Declaration of Helsinki.

### Variables and measurement

Psychometric tools were developed based on a review of prior studies to identify relevant variables and were modified to fit the study area [23–25]. The questionnaire comprised several parts (see S1 Questionnaire or S2 Questionnaire). Part 1 captured demographic and social details, such as sex, age, marital status, education level, alcohol consumption, and smoking status. Part 2 captured participants’ knowledge of noncommunicable diseases (NCDs), including hypertension, diabetes mellitus, hyperlipidemia, coronary heart disease, and kidney disease [23,26]. The questionnaire was designed to evaluate participants’ awareness of the causes, symptoms, complications, risk behaviors, and abnormal conditions associated with these diseases. It comprised 25 items, each with three response options: “yes,” “no,” and “don’t know.” A “yes” response was coded as 1, whereas a “no” response was coded as 0; no “don’t know” responses were recorded. The total knowledge ranged from 0 to 25, with higher scores indicating greater knowledge of NCDs.

Part 3 captured self-management of NCDs and was adapted from the Health Education Division, Department of Health Promotion, Ministry of Public Health [24,27]. This questionnaire retrospectively assessed participants’ self-care behaviors during the preceding month, including dietary practices, medication adherence, physical activity, and stress management. It was adapted to suit the sociocultural context of the target population in the study area. The questionnaire comprised five domains food consumption, physical activity, medication use, stress management, and annual health assessment with a total of 25 items. The total score ranged from 25 to 100, with higher scores indicating better self-care behavior. Responses were rated according to the frequency of practice: more than five times per week (4 points), three to four times per week (3 points), one to two times per week (2 points), and no practice or no activity performed during that week (1 point). Part 4 measured HRQoL using the WHOQoL-Old) [25,28], which was adapted from previous research [29]. This instrument had previously been translated into Thai [29] and comprises 24 items, with a total score ranging from 24 to 120. It covers six domains: (1) sensory abilities (SAB; 4 items), (2) autonomy (AUT; 4 items), (3) past, present, and future activities (PPF; 4 items), (4) social participation (SOP; 4 items), (5) death and dying (DAD; 4 items), and (6) intimacy (INT; 4 items). Each item was rated on a five-point Likert scale: not at all (1 point), a little (2 points), a moderate amount (3 points), quite a lot (4 points), and an extreme amount (5 points). Higher scores indicated better health-related quality of life.

After completing the first draft of the questionnaire, it was checked for accuracy using item-objective congruence [30] and reviewed by three external experts in their respective fields (internal medicine, behavioral health, and public health). The questionnaire was validated using a sample of 30 elderly people with similar background characteristics. The Index of Item-Objective Congruence was used to evaluate content validity, and the result was 0.84. The Kuder–Richardson formula (KR20 = 0.79) was used to assess the questionnaire’s reliability. The Cronbach’s alpha coefficients for Part 3 and Part 4 of the questionnaire were 0.80 and 0.81, respectively. Composite scores were created for all scales by adding the relevant items, and larger scores signified better knowledge, self-management, and QoL.

### Statistical analysis

Data were analysed with SPSS version 29 and its inbuilt Hayes’ PROCESS Macro. In the exploratory analysis, the data were summarized, and missing observations were identified and dealt with using descriptive statistics (i.e., frequency and averages). Furthermore, assumptions governing multiple linear regression analyses (i.e., linearity of the relationships, independence of errors around the regression line, the absence of multicollinearity, and normal distribution of the variables) were assessed in harmony with previous research [31,32].

The results of the “Missing Completely at Random” test at p > 0.05 suggested that the missing data were randomly distributed and constituted less than 10% of the data. This outcome allowed us to deal with missing data using the “multiple imputation” method [33], a procedure that involves plausible imputation of data based on standard statistical distributions. Linearity of the relationships was assessed with curve estimation following previous research [34]. This procedure evaluates whether the relationships fit a linear function and compares a linear function with several other non-linear relationships. The result showed that a linear function best fitted the data, and Fig 1 is a sample of charts from curve estimation.

**Fig 1:**
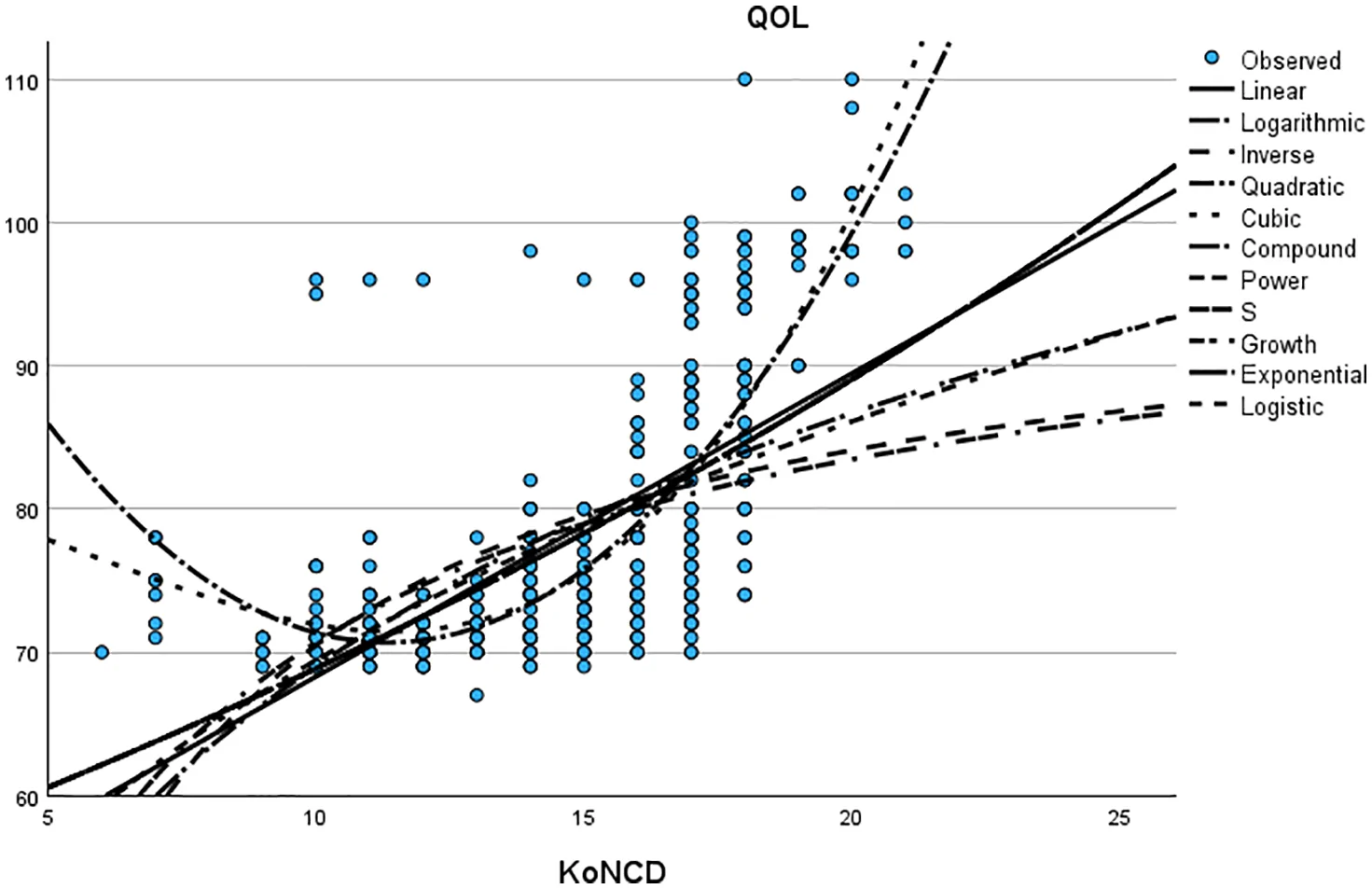
A sampled chart from the curve estimation of the relationships. Note: QoL – quality of life, KoNCD – knowledge of NCDs.

Normal distribution of the data was confirmed with box plots showing no outliers and a Kolmogorov-Smirnov test with non-significant results at p > 0.05. This procedure followed the recommendations in the literature [32]. Following recommendations in the literature [32], the independence of errors around the regression line was confirmed with a scatter diagram showing no pattern. The diagram was a plot of residuals against predicted values from the moderated mediation model. Finally, the absence of multicollinearity was confirmed with tolerance values ≥0.5 for each independent variable [31,32]. In the final phase of data analysis, we fitted the moderated mediation model with Hayes’ PROCESS Macro (Model 7, see Fig 2). The non-adjusted model did not include the confounding variables (i.e., Age, gender, marital status, education, smoking status, number of NCDs, and whether the individual drank alcohol), whereas the adjusted model incorporated the covariates. Estimations were based on 5000 bias-corrected sampling iterations and a 95% confidence interval. Centering and the computation of interaction effects were automatically set in Hayes’ PROCESS Macro. The statistical significance of the results was set at a minimum of p < 0.05.

**Fig 2:**
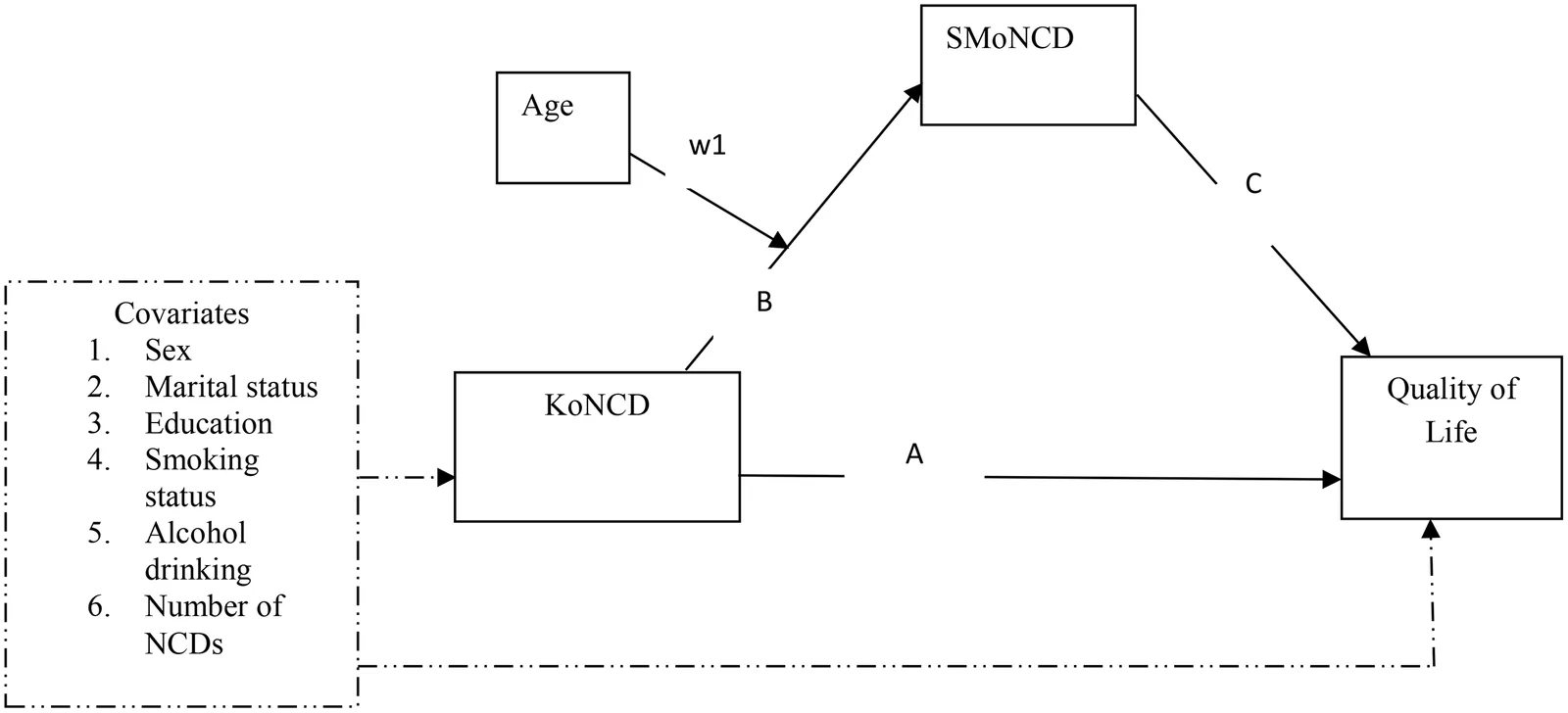
Authors’ conceptualization of the moderated mediation. Note: NCDs – non-communicable diseases; SMoNCD – self-management of NCDs, KoNCD – knowledge of NCDs; QoL – quality of life; broken arrows represent potential confounding; A – association of knowledge of NCDs with QoL; B – association of knowledge of NCDs with self-management of NCDs; C – association of self-management of NCDs with QoL; w1 – age as a moderator of the association of knowledge of NCDs with self-management of NCDs; and M – self-management of NCDs as a mediator of the association of knowledge of NCDs with QoL.

## Results

### Summary statistics

About 58% (n = 244) of the participants were women, whereas the average age of the participants was about 70 years (Mean = 69.65; SD = 5.73). The average knowledge of NCDs was 15 (Mean = 14.75; SD = 2.92), and the average for self-management of NCDs was 77 (Mean = 77.20; SD = 7.99). The average number of NCDs in the sample was about 2 (Mean = 1.69; SD = 0.691), and the number of NCDs ranged from 1 to 4, as shown in Table 1.

**Table 1:** Summary statistics of the participants (N = 424).

Variable	Group	n	%	Mean	SD	Min-Max
Categorical variables						
Sex	Male	180	42.45	---	---	---
	Female	244	57.55	---	---	---
Marital status	Single/divorced/widowed	123	29.01	---	---	---
	Married	301	70.99	---	---	---
Education	No	162	38.21	---	---	---
	Primary education	229	54.01	---	---	---
	Junior high school	33	7.78	---	---	---
Smoking status	No	353	83.25	---	---	---
	Yes	71	16.75	---	---	---
Alcohol intake	No	221	52.12	---	---	---
	Yes	203	47.88	---	---	---
	Total	424	100.00	---	---	---
Continuous variables						
Age (yrs)	---	---	---	69.65	5.73	60-88
NoNCDs	---	---	---	1.69	0.691	1-4
KoNCD	---	---	---	14.75	2.92	6-21
SMoNCD	---	---	---	77.20	7.99	14-119
QoL	---	---	---	65.28	8.06	56-92

Note: n – frequency, % - percent, SD – standard deviation, Min – minimum, Max – maximum, KoNCD – knowledge of non-communicable diseases; SMoNCD – self-management of non-communicable diseases, QoL – quality of life.

### Bivariate correlations

There was a strong positive correlation between knowledge of NCDs and self-management of NCDs (r = 0.644, p < 0.001, two-tailed) as well as QoL (Table 2; r = 0.639, p < 0.001, two-tailed). Thus, older adults with higher scores of knowledge of NCDs reported larger scores of self-management of NCDs and QoL. Self-management of NCDs was positively correlated with QoL (r = 0.701, p < 0.001, two-tailed), suggesting that larger scores of self-management of NCDs were associated with higher QoL. Age was negatively correlated with both knowledge of NCDs (r = −0.368, p < 0.001, two-tailed) and self-management of NCDs (r = −0.344, p < 0.001, two-tailed). This result suggests that scores of knowledge and self-management of NCDs were lower in older age.

**Table 2:** Zero-order bivariate correlations of the variables.

	1	2	3	4	5	6	7	8	9	10
1. KoNCD	1	.644**	.639**	−.368**	0.051	0.089	.461**	−.231**	−0.082	0.056
2. SMoNCD		1	.701**	−.344**	0.061	0.082	.311**	−.239**	−.131**	0.042
3. QoL			1	−.419**	.100*	0.069	.259**	−.323**	−0.081	.101*
4. Age (yrs)				1	0.093	−.287**	−.403**	0.001	0.009	−.144**
5. Female (ref – male)					1	−0.065	−.106*	−.167**	−.433**	−.323**
6. Married (ref – not married)						1	.193**	0.07	0.008	0.072
7. Educated (ref – no education)							1	0.052	−0.011	0.054
8. NoNCDs								1	.099*	−0.019
9. Smoker (ref – non-smoker)									1	.240**
10. Drinks alcohol (ref – no)										1

**p < 0.001; *p < 0.05; NoNCDs – number of non-communicable diseases; KoNCD – knowledge of non-communicable diseases; SMoNCD – self-management of non-communicable diseases, QoL – quality of life.

### Direct and indirect associations

After adjusting for the covariates (see Table 3), the study found a positive association of knowledge of NCDs with QoL (β = 0.31, p < 0.001; two-tailed). Knowledge of NCDs was positively associated with self-management of NCDs (β = 0.60, p < 0.001; two-tailed). Self-management of NCDs was also positively associated with QoL (β = 0.46, p < 0.001; two-tailed), confirming that larger scores of self-management of NCDs were associated with larger scores of QoL. Notably, the indirect association of knowledge of NCDs with QoL through self-management of NCDs was significant (β = 0.28, p < 0.001; two-tailed). Thus, self-management of NCDs transmitted the influence of knowledge of NCDs to QoL.

**Table 3:** Direct and indirect regression coefficients (N = 424).

Dependent variable	Path	Independent variable	Unstandardised Coeff (B)	Beta (β)	SE (of B)	t	95% CI (of B)		Indirect effects			
											95% CI (of β)	
							Lower Bound	Upper Bound	β	SE	Lower Bound	Upper Bound
Non-adjusted model												
QoL	<---	KoNNCD	1.060	0.320***	0.141	7.495	0.782	1.338	0.319***	0.066	0.212	0.446
QoL	<---	SMoNCD	0.598	0.494***	0.052	11.570	0.497	0.700				
SMoNDC	<---	KoNNCD	2.115	0.639***	0.124	17.053	1.871	2.359				
Adjusted model												
QoL	<---	KoNNCD	1.023	0.309***	0.147	6.952	0.734	1.313	0.275***	0.058	0.184	0.394
QoL	<---	SMoNCD	0.559	0.462***	0.051	11.027	0.459	0.658				
SMoNDC	<---	KoNNCD	1.630	0.596***	0.118	13.826	1.398	1.862				
Covariates (From the adjusted model)												
QoL	<---	Age (yrs)	-0.324	-0.192***	0.062	-5.195	-0.447	-0.202				
QoL	<---	Female (ref – male)	1.913	0.098**	0.713	2.682	0.511	3.315				
QoL	<---	Married (ref – not married)	-0.701	-0.033	0.703	-0.997	-2.082	0.681				
QoL	<---	Educated (ref – no education)	-1.806	-0.091*	0.744	-2.427	-3.268	-0.343				
QoL	<---	Smoker (ref – non-smoker)	0.826	0.032	0.916	0.902	-0.975	2.628				
QoL	<---	Drinks alcohol (ref – no)	1.303	0.067*	0.654	1.993	0.018	2.588				
QoL	<---	NoNCDs	-1.165	-0.101*	0.448	-2.600	-2.048	-0.284				
SMoNDC	<---	Age (yrs)	-0.426	-0.253***	0.070	-6.074	-0.564	-0.288				
SMoNDC	<---	Female (ref – male)	2.014	0.103*	0.810	2.486	0.421	3.606				
SMoNDC	<---	Married (ref – not married)	-0.746	-0.035	0.798	-0.934	-2.315	0.823				
SMoNDC	<---	Educated (ref – no education)	-1.945	-0.098*	0.845	-2.302	-3.606	-0.284				
SMoNDC	<---	Smoker (ref – non-smoker)	-0.131	-0.005	1.036	-0.126	-2.167	1.906				
SMoNDC	<---	Drinks alcohol (ref – no)	1.430	0.074	0.742	1.926	-0.029	2.889				
SMoNDC	<---	NoNCDs	-1.919	-0.137***	0.461	-4.162	-2.825	-1.013				

***p < 0.001; **p < 0.01; *p < 0.05; KoNCD – knowledge of non-communicable diseases; SMoNCD – self-management of non-communicable diseases, QoL – quality of life; NoNCDs – number of non-communicable diseases; CI – confidence interval; SE – standard error; Coeff – coefficient.

### Moderated mediation

In Table 4, age was negatively associated with self-management of NCDs (β = −0.28, p < 0.001; two-tailed). Thus, the ability to self-manage NCDs declined with age. The interaction between age and knowledge of NCDs was negatively associated with QoL after adjusting for the covariates (β = −0.13, p < 0.001; two-tailed). Table 5 shows the conditional indirect effects and indices of moderated mediation from the moderated mediation model. The index of moderated mediation in the adjusted model is negative and significant (index = −0.075; p < 0.001). This index and the conditional indirect effects suggest that the positive association of knowledge of NCDs with QoL was weaker at older age. These results are depicted in Fig 3. Finally, the indirect association of knowledge of NCDs with QoL was weaker at older age (see indirect effects in Table 5).

**Table 4:** Coefficients from the moderated mediation model (N = 424).

Predictors	SMoNCD			QoL		
	*Coeff*	*SE*	*t*	*Coeff*	*SE*	*t*
	Adjusted					
Constant	75.430***	1.717	43.925	28.886***	4.371	6.609
KoNCD	1.461***	0.113	12.942	1.104***	0.151	7.317
SMoNCD	---	---	---	0.597***	0.052	11.569
Age (yrs)	−0.275***	0.057	−4.787	---	---	---
AgexKoNCD	−0.125***	0.017	−7.561	---	---	---
*Covariates*						
Female (ref – male)	0.448	0.649	0.691	1.730*	0.734	2.355
Married (ref – not married)	0.119	0.639	0.186	0.140	0.705	0.199
Educated (ref – no education)	0.238	0.679	0.350	−0.877	0.744	−1.178
Smoker (ref – non-smoker)	−1.878	0.829	0.829	0.737	0.945	0.780
Drinks alcohol (ref – no)	0.536	0.595	0.901	1.637*	0.671	2.442
NoNCDs	−0.985*	0.422	−2.334	−1.803***	0.476	−3.785

***p < 0.001; *p < 0.05; ^---^not applicable; KoNCD – knowledge of non-communicable diseases; SMoNCD – self-management of non-communicable diseases, QoL – quality of life; NoNCDs – number of non-communicable diseases; SE – standard error; Coeff – coefficient.

**Table 5:** Conditional indirect effects and indirect effects based on 5000 bias-corrected bootstraps (N = 424).

	*Coeff*	95% CI		*Coeff*	95% CI	
		Lower	Upper		Lower	Upper
	Conditional indirect effect			Indirect effects		
Non-adjusted (IMM: Index = −0.073**; *SE* = 0.017)						
−1SD	2.075***	1.843	2.307	1.241***	0.815	1.755
Mean	1.588***	1.387	1.789	0.950***	0.628	1.348
+1SD	0.735***	0.422	1.048	0.440***	0.244	0.720
Adjusted (IMM: Index = −0.075**; *SE* = 0.018)						
−1SD	2.044***	1.801	2.286	1.221***	0.820	1.733
Mean	1.543***	1.324	1.762	0.922***	0.619	1.312
+1SD	0.666***	0.334	0.998	0.398***	0.217	0.659

***p < 0.001; **p < 0.01; IMM – index of moderated mediation; CI confidence interval; SE – standard error; Coeff – coefficient (unstandardized); SD – standard deviation.

**Fig 3:**
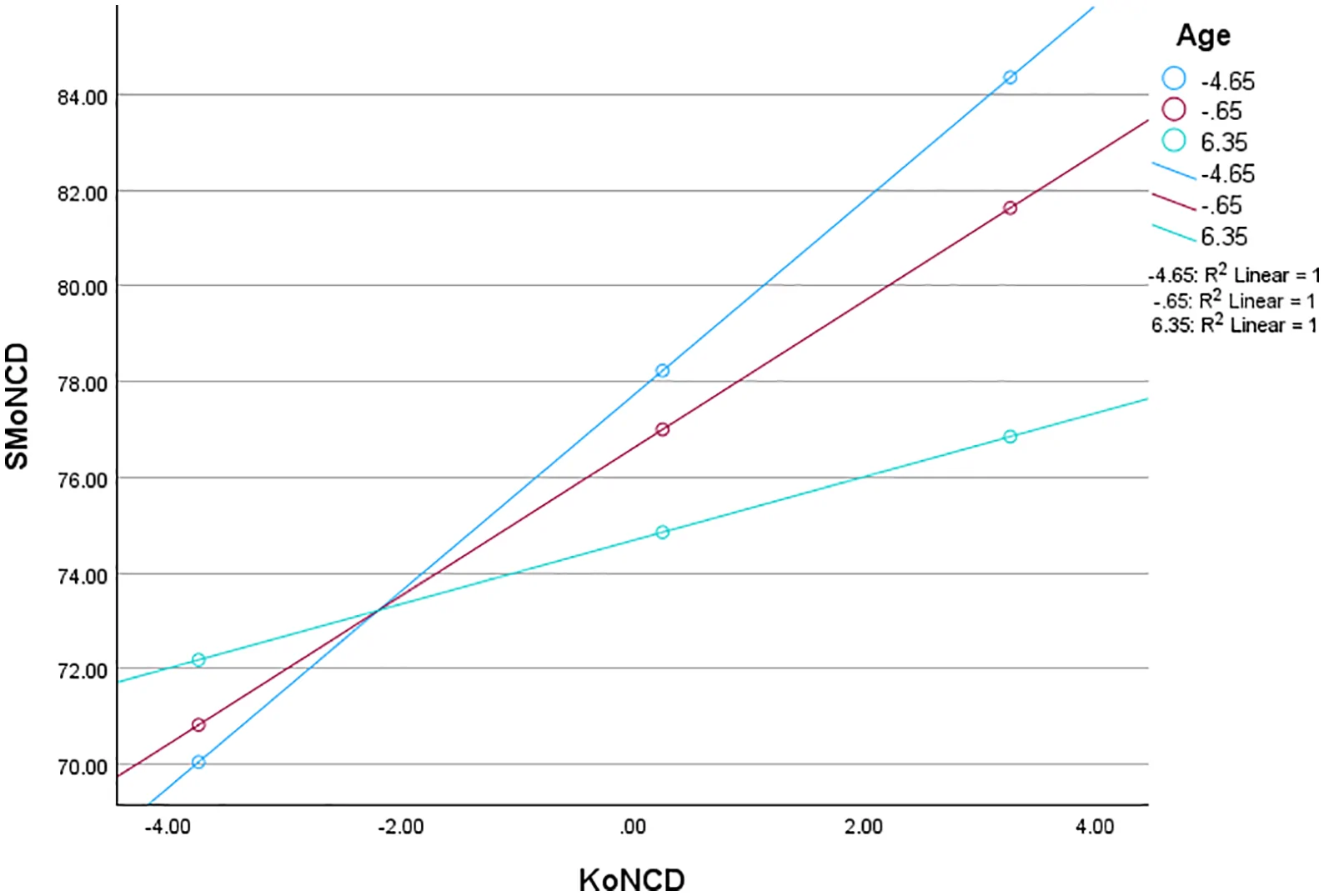
The association of knowledge of NCDs with self-management of NCDs at different ages (N = 424).

## Discussion

This study investigated whether the indirect association between knowledge of NCDs and QoL through self-management of NCDs was moderated by age. It focused on older adults living in rural communities in Phayao, Northern Thailand.

The findings suggest that greater knowledge of NCDs enhances the ability of older adults to manage their chronic diseases effectively. Specifically, individuals with higher levels of NCD-related knowledge are more likely to engage in appropriate dietary control, maintain regular physical activity, and adhere to prescribed medication regimens. These self-management practices, in turn, contribute to improved disease control and overall QoL. These findings are consistent with the concept of self-management, which encompasses behaviors individuals adopt to maintain and promote their health and well-being. Effective self-management is grounded in three fundamental components: knowledge, skills, and motivation [35,36]. Moreover, a growing body of evidence has demonstrated that health knowledge plays a crucial role in shaping health-related behaviors. Individuals with a better understanding of their diseases and appropriate self-care strategies are more likely to make informed decisions and engage in behaviors that support disease control and overall QoL [35,37]. The foregoing evidence aligns with previous studies that emphasize that knowledge, comprehension, and analytical thinking play a pivotal role in shaping appropriate health-related behaviors [38]. Therefore, implementing strategies that foster continuous learning and strengthen the connection between knowledge of NCDs and HRQoL is crucial [3,21]. This study further identifies age-related disparities in self-management in rural settings. These findings provide a basis for developing more precisely targeted interventions. In line with prior research that has advocated for national strategies that integrate multisectoral networks to enhance health literacy on NCDs among older adults [21], this study further identifies age-related disparities in self-management in rural settings. These findings provide a basis for developing more precisely targeted interventions.

This study further confirmed a significant relationship between knowledge of NCDs and QoL among older adults. Knowledge of NCDs influences QoL indirectly through the mechanism of self-management. The moderated mediation analysis further revealed that this indirect relationship is weaker in older age. Thus, the likelihood that self-management of NCDs would transmit a positive influence from knowledge of NCDs to QoL is lower in older age groups. This evidence is rooted in the fact that knowledge of NCDs is associated with poorer self-management of NCDs in older age groups. To explain, physiological changes, such as a decline in physical functional ability, reduced cognitive capacity, and slower responsiveness to external stimuli, are outcomes of ageing [39], and these changes can impair health information processing, disease comprehension, and self-management abilities [39], giving rise to a weaker association between knowledge and self-management of NCDs in old age.

The above findings are consistent with prior research that demonstrated that age significantly influences self-management ability among older adults with chronic diseases. Increasing age is negatively associated with self-management capacity [15], a trend supported by previous studies showing that older patients often experience a diminished ability to manage their conditions effectively [40–42]. Self-care competencies here refer to measured self-management behaviors (dietary, medication, exercise, and stress related), which are directly relevant to NCD self-management outcomes [12,15]. This decline may result from age-related deterioration in physical functioning or the cumulative effects of chronic illness, both of which can hinder the capacity to engage in consistent and effective self-care practices [40]. Furthermore, previous reviews have highlighted that differences in family and community contexts influence how older adults acquire and practice health behaviors, resulting in diverse patterns of self-management [35,36]. Therefore, relevant organizations and primary healthcare professionals should prioritize strengthening the self-management capacity among older adults by providing context-appropriate support in key areas, such as physical activity, nutrition, and disease management, and other health-promoting activities suited to local needs and environments.

The results further underscore the need to improve self-care competencies among older adults. In particular, greater attention should be given to improving dietary control, regular physical activity, medication adherence, and stress management, as these aspects continue to need development and support. This recommendation aligns with the theoretical perspective that self-management in individuals with chronic diseases extends beyond compliance with medical advice and involves an active, participatory process to maintain health [39]. Effective self-management encompasses three core components: medical management, which includes proper medication adherence and symptom monitoring; role management, which involves adaptation to disease-related limitations in daily life; and emotional management, which focuses on coping with stress and regulating emotional well-being [11]. Although older adults generally possess basic knowledge of chronic diseases, they often lack an in-depth understanding of potential complications and medication side effects [43]. This aligns with previous reviews, which emphasize that effective promotion of self-management requires support from a patient-centered healthcare system [12,43]. Such an approach should provide continuous education, skills training, and motivational reinforcement to empower patients to actively maintain and improve their own health [12].

This study highlights that knowledge regarding NCDs plays a crucial role in promoting self-management and enhancing QoL among older adults living in rural areas of Phayao Province. The effect is particularly evident among individuals who retain the capacity to learn and modify their health-related behaviors. However, the relationship between knowledge and self-management tends to diminish with advancing age, reflecting the limitations in learning ability and behavioral adaptability among older elderly populations. Consistent with the concept of individualized behavior change, this approach is recognized as an effective framework for promoting self-management. It emphasizes a process by which individuals can realistically assess their own knowledge, skills, and abilities; set achievable goals; monitor their progress; build motivation; and respond appropriately to feedback under the guidance of healthcare professionals [7,44]. Previous studies have reported that individuals with a good level of knowledge regarding NCDs tend to engage in appropriate health behaviors and are better able to reduce the risk of complications [45,46]. This finding indicates that knowledge serves as a fundamental basis for self-care and is positively associated with self-management among patients with chronic non-communicable diseases [47]. Therefore, proactive educational promotion strategies are crucial for enhancing the capacity of older adults to engage in sustained self-management and adopt appropriate, long-term health behaviors [48]. This is particularly important in rural areas where access to healthcare services is limited. Implementing such strategies can help reduce the risk of developing NCDs and promote long-term improvements in QoL among older adults. The design of health promotion programs should take into account the learning capacity of each older adult, employing participatory learning processes that emphasize feasible goal setting, progress monitoring, and appropriate feedback responses. In addition, fostering intrinsic motivation is essential to strengthen self-regulatory capacity. Integrating these concepts into primary healthcare services can enhance the effectiveness and sustainability of self–health management among older adults.

This study highlights that knowledge regarding NCDs plays a crucial role in promoting self-management and enhancing the quality of life among older adults living in rural areas of Phayao Province. The effect is particularly evident among individuals who retain the capacity to learn and modify their health-related behaviors. However, the relationship between knowledge and self-management tends to diminish with advancing age, reflecting the limitations in learning ability and behavioral adaptability among older elderly populations. Consistent with the concept of Individualized Behavior Change, this approach is recognized as an effective framework for promoting self-management. It emphasizes a process in which individuals realistically assess their own knowledge, skills, and abilities; set achievable goals; monitor their progress; build motivation; and respond appropriately to feedback under the guidance of healthcare professionals [7,44]. Previous studies have reported that individuals with a good level of knowledge regarding NCDs tend to engage in appropriate health behaviors and are better able to reduce the risk of complications [45,46]. This finding indicates that knowledge serves as a fundamental basis for self-care and is positively associated with self-management among patients with chronic non-communicable diseases [47]. Therefore, proactive educational promotion strategies are crucial for enhancing the capacity of older adults to engage in sustained self-management and adopt appropriate, long-term health behaviors [48]. This is particularly important in rural areas where access to healthcare services is limited. Implementing such strategies can help reduce the risk of developing NCDs and promote long-term improvements in the quality of life among older adults. The design of health promotion programs should take into account the learning capacity of each older adult, employing participatory learning processes that emphasize feasible goal setting, progress monitoring, and appropriate feedback responses. Additionally, fostering intrinsic motivation is essential to strengthen self-regulatory capacity. Integrating these concepts into primary healthcare services can enhance the effectiveness and sustainability of self-health management among older adults.

### Limitations

This study has some limitations. First, the cross-sectional design restricts the ability to draw causal inferences. Future research should therefore employ experimental and longitudinal designs that enable the monitoring of changes in self-management behaviors and QoL in response to an intervention. Several variables (e.g., healthcare access, disease severity, functional status, and social support) that could confound the relationships were not measured and included in our analysis. Future researchers may consider these and any other relevant confounding variables. The inclusion of in-depth assessments of self-management skills would yield a more comprehensive and nuanced analysis of the mechanisms behind the confirmed relationships. The representativeness of the sample is limited by the use of convenience sampling in a specific rural area of Northern Thailand, which may have resulted in selection bias. Therefore, the generalizability of the findings to other older adults with NCDs should be considered with caution. The unique social, cultural, and healthcare system characteristics of this region may differ from those in other areas. Future research may, therefore, utilise nationally representative samples of rural dwellers. Better still, future research may use the model tested in this study to compare rural and urban contexts, extending the scope of the evidence. Self-reported questionnaires were predominantly used in this study, which makes the study vulnerable to response bias, including social desirability and recall bias. The study did not incorporate all potential confounders, so we encourage future researchers to consider as many covariates as possible. Physiological ageing and cognitive ageing were used to explain the hypothesized relationships but were not included as variables in this study.

However, this study was the first to assess the relationship between knowledge, self-management, and HRQoL among older rural dwellers in Thailand. Hence, it provides unique evidence and advances gerontology literature. The study’s adoption of a conditional process model, or moderated mediation, builds upon previous evidence and uniquely demonstrates how knowledge of NCDs correlates with self-management of NCDs and HRQoL in older age. This analysis unfolds the implications of increasing age in a rural context on the self-management of NCDs. It reveals the need for scalable investment in programmes aimed at empowering older age groups to manage their NCDs successfully. Finally, it serves as a model for future experimental designs aimed at establishing causal inference through the conditional process model.

## Conclusions

Knowledge of NCDs was associated with better self-management of NCDs and QoL among older adults with chronic conditions in rural Northern Thailand. The positive association between knowledge of NCDs and the self-management of NCDs was weaker at older age. This outcome formed the basis for negative mediated mediation, which implies that the indirect association of knowledge of NCDs with QoL through the self-management of NCDs was weaker in older age. Thus, knowledge of NCDs is associated with better management of NCDs and QoL, but this relationship is weaker in older age. This evidence suggests the need for interventions aimed at enhancing both knowledge and self-management of NCDs in older age. To sustainably improve QoL among older adults with chronic conditions, public health agencies should implement proactive strategies at the primary care level. Such strategies should prioritize the development of self-management skills—including those related to dietary regulation, regular physical activity, appropriate medication adherence, and stress management particularly among the oldest age groups. In addition, the integration of patient-centered care, which emphasizes shared decision-making and active participation in health planning, is essential for empowering older adults to manage their NCDs.

## Supporting information

S1 Questionnaire Questionnaire in the Thai language. (DOCX)

S2 Questionnaire Questionnaire in the English language. (DOCX)

S1 File Dataset. (XLSX)
