# Supplementary material for: Knowledge of non-communicable diseases (NCDs) is less strongly associated with self-management of NCDs and quality of life in older age in Phayao, Thailand: A moderated mediation analysis
Source: PLoS One. 2026 Nov 11;21(11):e0362216. doi: 10.1371/journal.pone.0362216 (PMC13754156; doi:10.1371/journal.pone.0362216)
Supplement: Supplementary file 1 [file pone.0362216.s001.docx]

**แบบสอบถาม**

ID....................................

Name:………………………..

**ส่วนที่ 1** ลักษณะทางประชากรและสังคมของผู้สูงอายุ

**คำชี้แจง:** กรุณาทำเครื่องหมาย ( / ) ในช่องหรือเติมคำตอบที่ถูกต้องลงในช่องว่าง

1. เพศ ☐ (1) ชาย ☐ (2) หญิง
2. อายุ ……………………… ปี (กรุณาระบุให้ชัดเจน)
3. สถานภาพ

☐ (1) โสด/หย่าร้าง/หม้าย/แยกกันอยู่

☐ (2) สมรส	☐ (3) อื่น ๆ ………………………

4. ระดับการศึกษา

☐ (0) ไม่ได้เรียน	☐ (1) ประถมศึกษา

☐ (2) มัธยมศึกษาตอนต้น	☐ (3) มัธยมศึกษาตอนปลาย/ประกาศนียบัตรวิชาชีพ (ปวช.)

☐ (3) อื่น ๆ ………………………

5. ปัจจุบันท่านสูบบุหรี่หรือไม่

☐ (0) ไม่สูบ	☐ (1) สูบ

6. ปัจจุบันท่านดื่มแอลกอฮอล์หรือไม่

☐ (0) ไม่ดื่ม	☐ (1) ดื่ม

**ส่วนที่ 2 แบบสอบถามความรู้เกี่ยวกับโรคไม่ติดต่อเรื้อรัง (NCDs) ของผู้สูงอายุ**

**หมายเหตุ:** กรุณาทำเครื่องหมายในช่องคำตอบที่ตรงกับความเข้าใจ ความคิดเห็น และความเชื่อของท่านเกี่ยวกับโรคไม่ติดต่อเรื้อรังในข้อต่อไปนี้

ความหมายของคำตอบ

| **คะแนน** | **คำตอบ** | **ความหมาย** |
| --- | --- | --- |
| (1) | ใช่ | ท่านเห็นด้วย/ใช่กับข้อคำถาม |
| (0) | ไม่แน่ใจ | ท่านไม่แน่ใจเกี่ยวกับข้อคำถาม |
| (0) | ไม่ใช่ | ท่านไม่เห็นด้วย/ไม่ใช่กับข้อคำถาม |

| **ข้อ** | **ข้อความ** | **ใช่** | **ไม่แน่ใจ** | **ไม่ใช่** |
| --- | --- | --- | --- | --- |
| 1 | หากไม่ควบคุมความดันโลหิตในร่างกาย หัวใจจะทำงานหนัก |  |  |  |
| 2 | โรคความดันโลหิตสูงในระยะยาวจะทำให้ระบบไหลเวียนโลหิตตีบและแตก |  |  |  |
| 3 | ค่าดัชนีมวลกาย (BMI) ที่สูงมีความสัมพันธ์กับภาวะต่าง ๆ เช่น เบาหวานชนิดที่ 2 เนื่องจากร่างกายมีความต้องการอินซูลินเพิ่มขึ้น |  |  |  |
| 4 | ความดันโลหิตสูงที่ไม่ได้รับการรักษา จะทำให้ดวงตาเสื่อม |  |  |  |
| 5 | ความดันโลหิตตั้งแต่ 130/80 มิลลิเมตรปรอทขึ้นไป ถือว่าเป็นความดันโลหิตสูง |  |  |  |
| 6 | เหงื่อออก มือสั่น และใจสั่น เป็นสัญญาณของภาวะน้ำตาลในเลือดต่ำ |  |  |  |
| 7 | การตรวจระดับน้ำตาลในเลือดหลังรับประทานอาหาร 2 ชั่วโมง หากสูงกว่า 180 มก./ดล. แสดงว่าระดับน้ำตาลในเลือดสูง |  |  |  |
| 8 | ค่าน้ำตาลเฉลี่ยสะสมหรือฮีโมโกลบินเอวันซี (HbA1C) ของผู้ป่วยเบาหวานที่น้อยกว่า 8% ถือว่าปกติ |  |  |  |
| 9 | โรคเบาหวานเกิดจากการทำงานผิดปกติของตับอ่อน ทำให้ไม่สามารถควบคุมระดับน้ำตาลในเลือดของร่างกายได้ |  |  |  |
| 10 | ภาวะแทรกซ้อนของโรคเบาหวาน ได้แก่ ไตวายเรื้อรัง แผลหายช้า ชาปลายมือปลายเท้า และจอประสาทตาเสื่อมจากเบาหวาน |  |  |  |
| 11 | โรคเบาหวานและโรคความดันโลหิตสูงไม่ใช่สาเหตุของโรคไต |  |  |  |
| 12 | ไตมีหน้าที่ขับน้ำและกำจัดของเสียออกจากร่างกาย |  |  |  |
| 13 | ภาวะไตวายเรื้อรังเป็นภาวะที่ไตสูญเสียการทำงานเพียงชั่วคราว และสามารถกลับมาทำงานเป็นปกติได้ด้วยการรักษา |  |  |  |
| 14 | สาเหตุที่พบบ่อยของภาวะไตวายเรื้อรัง ได้แก่ โรคเบาหวาน โรคความดันโลหิตสูง กรวยไตอักเสบเรื้อรัง และนิ่วในไต |  |  |  |

| **ข้อ** | **ข้อความ** | **ใช่** | **ไม่แน่ใจ** | **ไม่ใช่** |
| --- | --- | --- | --- | --- |
| 15 | อาการผิดปกติที่อาจพบในผู้ป่วยโรคไต ได้แก่ ใบหน้า รอบดวงตา หรือขาบวม และการปัสสาวะผิดปกติ เช่น ตื่นขึ้นมาปัสสาวะบ่อยในเวลากลางคืน |  |  |  |
| 16 | โรคหลอดเลือดสมองเกิดจากพันธุกรรม |  |  |  |
| 17 | ผู้ที่มีน้ำหนักตัวต่ำกว่าเกณฑ์หรือผอม มีความเสี่ยงต่อการเกิดโรคหลอดเลือดสมองมากกว่าผู้ที่มีน้ำหนักเกินหรือเป็นโรคอ้วน |  |  |  |
| 18 | ภาวะไขมันในเลือดสูงเป็นปัจจัยเสี่ยงของโรคหลอดเลือดสมอง |  |  |  |
| 19 | อาการปากเบี้ยว ลิ้นแข็ง และอาการชาหรืออ่อนแรงของแขนหรือขาข้างใดข้างหนึ่ง เป็นอาการของโรคหลอดเลือดสมอง |  |  |  |
| 20 | อาการพูดลำบาก พูดไม่ชัด หรือพูดไม่เป็นคำที่เข้าใจได้ ไม่ใช่สัญญาณเตือนของโรคหลอดเลือดสมอง |  |  |  |
| 21 | โรคเบาหวานเป็นปัจจัยเสี่ยงของโรคหลอดเลือดหัวใจ |  |  |  |
| 22 | โรคความดันโลหิตสูงไม่ใช่ปัจจัยเสี่ยงของโรคหลอดเลือดหัวใจ |  |  |  |
| 23 | ระดับคอเลสเตอรอลชนิด LDL (ไขมันชนิดไม่ดี) ตั้งแต่ 140 มิลลิกรัมต่อเดซิลิตรขึ้นไป ถือเป็นระดับไขมันปกติที่ไม่เพิ่มความเสี่ยงต่อโรคหลอดเลือดหัวใจ |  |  |  |
| 24 | ระดับไตรกลีเซอไรด์ตั้งแต่ 150 มิลลิกรัมต่อเดซิลิตรขึ้นไป ถือเป็นระดับไขมันปกติและไม่เพิ่มความเสี่ยงต่อโรคหลอดเลือดหัวใจ |  |  |  |
| 25 | อาการปวดบริเวณคอ ไหล่ และแขนทั้งสองข้าง โดยเฉพาะข้างซ้าย เป็นสัญญาณเตือนของโรคหลอดเลือดหัวใจ |  |  |  |

**ส่วนที่ 3 แบบสอบถามพฤติกรรมการจัดการตนเองเพื่อป้องกันโรคไม่ติดต่อเรื้อรังในผู้สูงอายุ**

**คำชี้แจง:** แบบสอบถามนี้ต้องการสอบถามข้อมูลเกี่ยวกับพฤติกรรมการดูแลสุขภาพ การปฏิบัติตน และกิจกรรมต่าง ๆ ที่ท่านได้ปฏิบัติในช่วง 1 เดือนที่ผ่านมา โดยแบ่งออกเป็น 5 ด้าน โปรดอ่านข้อความในแต่ละข้อ แล้วเลือกคำตอบที่ตรงกับพฤติกรรมการปฏิบัติของท่านมากที่สุดเพียง 1 คำตอบ โดยทำเครื่องหมาย (✓) ในช่องที่ตรงกับคำตอบของท่าน

คำจำกัดความของระดับความถี่ในการปฏิบัติ

| **คะแนน** | **ความหมาย** |
| --- | --- |
| (4) | ปฏิบัติมากกว่า 5 ครั้งต่อสัปดาห์ขึ้นไป |
| (3) | ปฏิบัติ 3-4 ครั้งต่อสัปดาห์ |
| (2) | ปฏิบัติ 1-2 ครั้งต่อสัปดาห์ |
| (1) | ไม่ได้ปฏิบัติหรือทำกิจกรรมใดๆ ในสัปดาห์นี้ |

| **ข้อความ** | **ระดับความถี่ในการปฏิบัติ** | | | |
| --- | --- | --- | --- | --- |
|  | **(4)** | **(3)** | **(2)** | **(1)** |
| **ด้านการรับประทาน** |  |  |  |  |
| 1. รับประทานอาหารเค็ม เช่น ปลาเค็ม ผักกาดดอง เต้าหู้ยี้ หรือเต้าเจี้ยว |  |  |  |  |
| 2. เติมเครื่องปรุงรสขณะรับประทานอาหาร เช่น เกลือ น้ำปลา น้ำปลาร้า ซีอิ๊วขาว และผงชูรส |  |  |  |  |
| 3. รับประทานอาหารแปรรูป เช่น ปลากระป๋อง กะหล่ำปลีดอง แกงกะทิ ปลาเค็ม ปลาแห้ง บะหมี่กึ่งสำเร็จรูป |  |  |  |  |
| 4. รับประทานอาหารทะเล เช่น หมึก กุ้ง หอยนางรม หอยแครง และปูทะเล |  |  |  |  |
| 5. รับประทานอาหารที่มีโปรตีน เช่น ปลา เนื้อสัตว์ และไข่ |  |  |  |  |
| 6. รับประทานขนมหวาน เช่น ขนมไทย (ฝอยทอง กล้วยบวชชี ขนมหวานต่าง ๆ) เค้ก ขนมปัง เบเกอรี่ ไอศกรีม ช็อกโกแลต |  |  |  |  |
| 7. รับประทานอาหารไขมันสูง เช่น หนังปลาทอดกรอบ แคบหมูหรือมันหมู เครื่องใน อาหารทอด ไข่ทอด ไข่เจียว อาหารเหนือ เช่น แกงฮังเล |  |  |  |  |
| 8. รับประทานผลไม้รสหวาน เช่น ทุเรียน มะม่วงสุก ขนุน และลำไย |  |  |  |  |
| 9. ดื่มเครื่องดื่มรสหวาน เช่น นมเย็น ชาเย็น กาแฟ น้ำผลไม้ น้ำอัดลม |  |  |  |  |
| 10. รับประทานอาหารที่หลากหลายครบ 5 หมู่ในแต่ละวัน วันละ 3 มื้อ |  |  |  |  |
| **ด้านการออกกำลังกาย** |  |  |  |  |
| 11. อบอุ่นร่างกายก่อนออกกำลังกาย 5-10 นาที โดยการยืดกล้ามเนื้อ |  |  |  |  |
| 12. ออกกำลังกายโดยใช้ยางยืดออกกำลังกาย เดินเร็ว หรือปั่นจักรยาน อย่างต่อเนื่อง 30-40 นาที |  |  |  |  |

| **ข้อความ** | **ระดับความถี่ในการปฏิบัติ** | | | |
| --- | --- | --- | --- | --- |
|  | **(4)** | **(3)** | **(2)** | **(1)** |
| **ด้านการออกกำลังกาย (ต่อ)** |  |  |  |  |
| 13. มีการเคลื่อนไหวร่างกายหรือทำกิจกรรมทางกายในงานบ้านหรืออาชีพ เช่น งานบ้าน/ทำสวน/ทำไร่/ทำนา หรือเดินไปทำงาน จนรู้สึกเหนื่อยปานกลาง อย่างน้อยครั้งละ 30 นาที |  |  |  |  |
| 14. มีกิจกรรมทางกายหรือกิจกรรมนันทนาการ เช่น กีฬาพื้นบ้าน รำวง หรือกิจกรรมที่มีจังหวะ |  |  |  |  |
| 15. สังเกตอาการผิดปกติของร่างกายขณะออกกำลังกาย เช่น หัวใจเต้นเร็วผิดปกติ แน่นหน้าอก หายใจลำบาก เวียนศีรษะ หน้ามืด |  |  |  |  |
| **ด้านการจัดการความเครียด** |  |  |  |  |
| 16. นอนหลับวันละ 7–9 ชั่วโมง |  |  |  |  |
| 17. สวดมนต์ นั่งสมาธิ ทำบุญ และกราบไหว้พระ |  |  |  |  |
| 18. ปรึกษาและพูดคุยกับสมาชิกในครอบครัว ญาติ หรือเพื่อน |  |  |  |  |
| 19. ซื้อยานอนหลับ วิตามินช่วยนอนหลับ หรือยาที่เกี่ยวข้องกับระบบประสาทมารับประทาน |  |  |  |  |
| 20. การควบคุมตนเอง จัดการอารมณ์โดยฝึกการหายใจเมื่อรู้สึกโกรธ หรือทำกิจกรรมร่วมกับครอบครัว เช่น รับประทานอาหาร ดูโทรทัศน์ หรือเดินทางท่องเที่ยว |  |  |  |  |
| **ด้านการรับประทานยา** |  |  |  |  |
| 21. อ่านฉลากด้านหน้าของบรรจุภัณฑ์ก่อนรับประทานยาทุกครั้ง |  |  |  |  |
| 22. รับประทานยาตามขนาดและปริมาณที่กำหนดไว้ในแผนการรักษา |  |  |  |  |
| 23. เมื่อลืมรับประทานยา ฉันรับประทานทันทีที่นึกได้ และเพิ่มขนาดยาเป็นสองเท่าในมื้อถัดไป |  |  |  |  |
| 24. ในช่วงเดือนที่ผ่านมา ฉันไม่ได้รับประทานยาตรงเวลา (เช่น เร็วหรือช้ากว่าเวลาปกติเกิน 1 ชั่วโมง) |  |  |  |  |
| 25. หากมีอาการผิดปกติจากการรับประทานยา ฉันจะไปพบแพทย์ทันที |  |  |  |  |

**ส่วนที่ 5 แบบสอบถามคุณภาพชีวิตของผู้สูงอายุ**

**คำชี้แจง:** แบบสอบถามนี้มีวัตถุประสงค์เพื่อประเมินคุณภาพชีวิตของผู้สูงอายุในช่วง 1 เดือนที่ผ่านมา โดยสอบถามความคิดเห็นและประสบการณ์เกี่ยวกับเรื่องต่าง ๆ ที่ระบุไว้ในแต่ละข้อ ซึ่งตรงกับความคิดเห็นหรือประสบการณ์ของท่านมากที่สุด โปรดทำเครื่องหมาย (/) ลงในช่องที่ตรงกับความคิดเห็นของท่านเพียง 1 ช่องต่อข้อ

| **คะแนน** | **ความหมาย** |  |
| --- | --- | --- |
| (5) | หมายถึง | มากที่สุด / เป็นประจำ |
| (4) | หมายถึง | มาก |
| (3) | หมายถึง | ปานกลาง |
| (2) | หมายถึง | น้อย |
| (1) | หมายถึง | ไม่เคย |

เกณฑ์การให้คะแนน

| **ข้อ** | **ข้อความ** | **ระดับการให้คะแนน** | | | | |
| --- | --- | --- | --- | --- | --- | --- |
|  |  | **1** | **2** | **3** | **4** | **5** |
| 1. | ความบกพร่องของประสาทสัมผัสส่งผลต่อชีวิตประจำวัน (การเสื่อมถอยหรือสูญเสียการทำงานของประสาทสัมผัส เช่น การได้ยิน การมองเห็น การได้กลิ่น การรับรส การสัมผัส ส่งผลต่อคุณภาพชีวิตของท่านอย่างไร) |  |  |  |  |  |
| 2. | การสูญเสียความสามารถของประสาทสัมผัสส่งผลต่อการเข้าร่วมกิจกรรม (การเสื่อมถอยหรือสูญเสียการทำงานของระบบประสาทสัมผัส เช่น การได้ยิน การมองเห็น การได้กลิ่น การรับรส หรือการสัมผัส ส่งผลต่อความสามารถ ความต้องการ หรือการเข้าร่วมกิจกรรมของชุมชน) |  |  |  |  |  |
| 3. | อิสระในการตัดสินใจด้วยตนเอง (ท่านมีอิสระในการตัดสินใจด้วยตนเอง เช่น การตัดสินใจว่าจะไปที่ใดหรือทำกิจกรรมอะไร มากน้อยเพียงใด) |  |  |  |  |  |
| 4. | การรู้สึกว่าควบคุมอนาคตของตนเองได้ (ท่านรู้สึกว่าสามารถควบคุมและวางแผนอนาคตของตนเองได้มากน้อยเพียงใด) |  |  |  |  |  |
| 5. | คนรอบข้างเคารพในอิสระของท่าน (ท่านรู้สึกว่าคนรอบข้างเคารพการตัดสินใจ ความเป็นอิสระทางความคิด และการตัดสินใจของท่านมากน้อยเพียงใด) |  |  |  |  |  |
| 6. | ความกังวลเกี่ยวกับลักษณะการเสียชีวิตของตนเอง  (ท่านกังวลเกี่ยวกับช่วงเวลาสุดท้ายของชีวิตมากน้อยเพียงใด) |  |  |  |  |  |

| **ข้อ** | **ข้อความ** | **ระดับการให้คะแนน** | | | | |
| --- | --- | --- | --- | --- | --- | --- |
|  |  | **1** | **2** | **3** | **4** | **5** |
| 7. | ความกลัวว่าจะไม่สามารถควบคุมการตายของตนเองได้  (ท่านกังวลว่าจะไม่สามารถควบคุมการตายของตนเองได้มากน้อยเพียงใด) |  |  |  |  |  |
| 8. | ความกลัวต่อการตาย  (ท่านกลัวความเป็นไปได้ที่จะเสียชีวิตมากน้อยเพียงใด) |  |  |  |  |  |
| 9. | ความกลัวความเจ็บปวดก่อนตาย  (ท่านกลัวความเจ็บปวดก่อนเสียชีวิตมากน้อยเพียงใด) |  |  |  |  |  |
| 10. | ปัญหาของการทำงานของประสาทสัมผัสส่งผลต่อความสามารถในการปฏิสัมพันธ์กับผู้อื่น  (ระบบประสาทสัมผัส/การรับสัมผัส เช่น การได้ยินลดลง การมองเห็นพร่ามัว การได้กลิ่นผิดปกติ การรับรสเปลี่ยนไป การสัมผัสผิดปกติ ส่งผลต่อการมีปฏิสัมพันธ์กับผู้อื่นของท่านมากน้อยเพียงใด) |  |  |  |  |  |
| 11. | สามารถทำสิ่งที่ท่านอยากทำได้ (ท่านสามารถทำหรือเข้าร่วมกิจกรรมที่ท่านสนใจได้มากน้อยเพียงใด) |  |  |  |  |  |
| 12. | ความพึงพอใจต่อโอกาสในการประสบความสำเร็จอย่างต่อเนื่อง (ท่านพึงพอใจกับโอกาสที่ได้รับ ซึ่งนำไปสู่ความสำเร็จในชีวิตมากน้อยเพียงใด) |  |  |  |  |  |
| 13. | ได้รับการยอมรับตามที่ท่านควรได้รับในชีวิต (ท่านรู้สึกว่าตนเองควรได้รับการเห็นคุณค่าและการให้เกียรติในชีวิตมากน้อยเพียงใด) |  |  |  |  |  |
| 14. | มีสิ่งที่ต้องทำเพียงพอในแต่ละวัน (ท่านรู้สึกว่ามีสิ่งที่ทำให้ดำเนินชีวิตอย่างเพียงพอมากน้อยเพียงใด) |  |  |  |  |  |
| 15. | ความพึงพอใจต่อสิ่งที่ท่านประสบความสำเร็จในชีวิต (ท่านพึงพอใจกับความสำเร็จในชีวิตของท่านมากน้อยเพียงใด) |  |  |  |  |  |
| 16. | ความพึงพอใจต่อวิธีการใช้เวลา (ท่านพึงพอใจกับวิถีการดำเนินชีวิตของท่านมากน้อยเพียงใด) |  |  |  |  |  |
| 17. | ความพึงพอใจต่อระดับการทำกิจกรรม (ท่านพึงพอใจกับความสามารถในการเข้าร่วมกิจกรรมกับผู้อื่นมากน้อยเพียงใด) |  |  |  |  |  |
| 18. | ความพึงพอใจต่อโอกาสในการมีส่วนร่วมในชุมชน (ท่านพึงพอใจกับโอกาสที่ได้เข้าร่วมกิจกรรมต่าง ๆ ในชุมชนของท่านมากน้อยเพียงใด) |  |  |  |  |  |

| **ข้อ** | **ข้อความ** | **ระดับการให้คะแนน** | | | | |
| --- | --- | --- | --- | --- | --- | --- |
|  |  | **1** | **2** | **3** | **4** | **5** |
| 19. | ความสุขกับสิ่งที่รอคอยในอนาคต (ท่านมีความสุขกับสิ่งที่รอคอยในอนาคต เช่น การที่บุตรหลานสำเร็จการศึกษาหรือแต่งงาน มากน้อยเพียงใด) |  |  |  |  |  |
| 20. | การประเมินการทำงานของประสาทสัมผัส (ท่านคิดว่าการทำงานของประสาทสัมผัส/การรับรู้ของท่าน เช่น การได้ยิน การมองเห็น การได้กลิ่น การรับรส การสัมผัส อยู่ในระดับดีเพียงใด) |  |  |  |  |  |
| 21. | รู้สึกมีเพื่อนคู่คิดในชีวิต (ท่านรู้สึกว่ามีเพื่อน (และไม่โดดเดี่ยว) มากน้อยเพียงใด) |  |  |  |  |  |
| 22. | การได้สัมผัสความรักในชีวิต (ในชีวิตของท่าน ท่านเคยได้รับประสบการณ์ความรักมากน้อยเพียงใด) |  |  |  |  |  |
| 23. | โอกาสในการให้ความรัก (ในชีวิตของท่าน ท่านเคยมีโอกาสในการรักบุคคลอื่นหรือสิ่งอื่นมากน้อยเพียงใด) |  |  |  |  |  |
| 24. | โอกาสในการได้รับความรัก (ท่านได้รับความรักจากบุตรหลานของท่านมากน้อยเพียงใด) |  |  |  |  |  |
