# Supplementary material for: Knowledge of non-communicable diseases (NCDs) is less strongly associated with self-management of NCDs and quality of life in older age in Phayao, Thailand: A moderated mediation analysis
Source: PLoS One. 2026 Nov 11;21(11):e0362216. doi: 10.1371/journal.pone.0362216 (PMC13754156; doi:10.1371/journal.pone.0362216)
Supplement: Supplementary file 2 [file pone.0362216.s002.docx]

**Questionnaire**

ID....................................

Name:………………………..

**Part 1** Demographic and social characteristics of the elderly

**Instructions:** Please put a check mark ( / ) in the box or fill in the blank with the correct answer.

General characteristics

1. Sex	(1) Male	🗌 (2) Female

2. Age…………………………………years (Please be specific)

3.Status

🗌 (1) Single/ divorced/widowed/separated

🗌 (2) Married

🗌 (3) Other…………………………

4. Education level

🗌 (0) No	🗌 (1) Primary education

🗌 (2) Junior high school

🗌 (3) Senior high school/Vocational certificate

5. Do you currently smoke

🗌 (0) No	🗌 (1) Yes

6. Do you currently drink alcohol

🗌 (0) No	🗌 (1) Yes

**Part 2: Questionnaire about the NCDs knowledge of the elderly.**

Note: Please marked 🗸in the answers that correspond to understanding, your opinions and beliefs about non -communicable diseases in the following joints.

|  | Definition | |  |  |  | | |  |  |
| --- | --- | --- | --- | --- | --- | --- | --- | --- | --- |
|  | (1) | | Yes | Meaning | You agree/yes with questions | | |  |  |
|  | (0) | | Unsure | Meaning | You are not sure about the question | | |  |  |
|  | (0) | | No | Meaning | You do not agree/not with the question | | |  |  |
| **Item** | | **Knowledge about NCDs** | | | | **Yes** | **Unsure** | | **No** |
| 1 | | If not maintaining the blood pressure in the body. The heart will work hard. | | | |  |  | |  |
| 2 | | Long-term hypertension disease will cause the circulatory system to narrow and break. | | | |  |  | |  |
| 3 | | Higher BMI levels are associated with conditions such as type 2 diabetes due to the body's increased demand for insulin | | | |  |  | |  |
| 4 | | High blood pressure is not treated, will cause the eyes to deteriorate. | | | |  |  | |  |
| 5. | | It is considered high blood pressure if the reading is 130/80 mmHg. | | | |  |  | |  |
| 6. | | Sweating, essential tremor, and heart palpitations are signs of low blood sugar. | | | |  |  | |  |
| 7. | | Blood sugar test 2 hours after eating. If it is higher than 180 mg/dl, it means your blood sugar level is high. | | | |  |  | |  |
| 8. | | The average blood sugar level or hemoglobin A1C (HbA1C) of diabetic patients is less than 8% which is normal. | | | |  |  | |  |
| 9. | | Diabetes is caused by abnormal functioning of the pancreas, which makes it unable to control the body's blood sugar levels. | | | |  |  | |  |
| 10. | | Complications of diabetes include chronic renal failure, slow wound healing, numbness in the extremities, and diabetic retinopathy. | | | |  |  | |  |
| 11. | | Diabetes and hypertension are not causes of kidney disease. | | | |  |  | |  |
| 12. | | The kidneys function to excrete water and remove waste from the body. | | | |  |  | |  |
| 13. | | Chronic kidney failure is a temporary loss of kidney function that can be reversed with treatment. | | | |  |  | |  |
| 14. | | Common causes of chronic renal failure include diabetes, hypertension, chronic pyelonephritis, and kidney stones. | | | |  |  | |  |
| 15. | | Abnormal symptoms that can be found in patients with kidney disease include swelling of the face, eyes or legs, and abnormal urination, such as getting up frequently to urinate at night. | | | |  |  | |  |
| 16. | | Stroke is caused by genetics. | | | |  |  | |  |
| 17. | | Underweight or thin is more at risk for stroke than overweight or obese. | | | |  |  | |  |
| 18. | | Hyperlipidemia is a risk factor for cerebrovascular disease. | | | |  |  | |  |
| 19. | | A crooked mouth, a stiff tongue, and numbness or weakness of one arm or leg on one side are symptoms of cerebrovascular disease. | | | |  |  | |  |

| **Item** | **Knowledge about NCDs** | **Yes** | **Unsure** | **No** |
| --- | --- | --- | --- | --- |
| 20. | Difficulty speaking, slurred speech, or incomprehensible speech are not warning signs of a stroke. |  |  |  |
| 21. | Diabetes is a risk factor for coronary heart disease. |  |  |  |
| 22. | Hypertension is not a risk factor for coronary heart disease. |  |  |  |
| 23. | Cholesterol levels (LDL cholesterol - bad fat) ≥140 md/dl is a normal fat level that does not put you at risk of coronary heart disease. |  |  |  |
| 24. | Triglyceride levels ≥150 mg/dl are considered normal fat levels and do not pose a risk for coronary heart disease. |  |  |  |
| 25. | Pain in the neck, shoulders, and both arms, especially the left side, is a warning sign of coronary artery disease. |  |  |  |

**Part 3: Questionnaire on self-management behaviors for prevention of non-communicable diseases among the elderly.**

Note: The questionnaire requests information on your health-care behavior, practices, and activities over the course of the previous month in five components. Please read the text box corresponding to your conduct and either answer only one question or put a checkmark (/) in it.

| **Definition** |  |  |
| --- | --- | --- |
| (4) | Meaning | Practice ≥ to 5 times/week or more. |
| (3) | Meaning | Practice 3-4 times / week |
| (2) | Meaning | Practice 1-2 times/week |
| (1) | Meaning | Never practiced or done anything |

| **Self-management behaviors** | **≥ 5 times/week or more (4)** | **3-4 times / week (3)** | **1-2**  **times/ week (2)** | **Never practiced**  **(1)** |
| --- | --- | --- | --- | --- |
| **In terms of eating** |  |  |  |  |
| 1. Eat salty foods such as salted fish, pickled mustard greens, fermented bean curd, or soybean paste. |  |  |  |  |
| 2. Add seasonings when eating, such as salt, fish sauce, fermented fish sauce, light soy sauce, and monosodium glutamate to food. |  |  |  |  |
| 3. Eating processed foods, such as canned fish, pickled cabbage, coconut milk curry, salted fish, dried fish, instant noodles. |  |  |  |  |
| 4. Eat seafood such as squid, shrimp, oysters, cockles, and sea crabs. |  |  |  |  |
| 5. Eat foods that contain protein such as fish, meat and eggs. |  |  |  |  |
| 6. Eat sweet desserts such as Thai desserts (Foi Thong, Kluai Buat Chee, sweetmeats, etc.), cakes, bread, bakery, ice cream, chocolate. |  |  |  |  |
| 7. Eating fatty foods such as crispy fried fish skin, pork snack or fat, offal, fried foods, fried beaten egg, omelette, northern food such as Hang Lay curry. |  |  |  |  |
| 8. Eat sweet fruits such as durian, ripe mango, jackfruit and longan. |  |  |  |  |
| 9. Sweet tasting drinks such as iced mil, iced tea, coffee, fruit juice, soft drinks. |  |  |  |  |
| 10. Eat a balanced diet with all 5 food groups in three meals a day. |  |  |  |  |
| **In terms of exercise** |  |  |  |  |
| 11. Warm up for 5-10 minutes before exercising by stretching your muscles. |  |  |  |  |
| 12. Exercise using resistance bands, brisk walking, or cycling continuously for 30-40 minutes. |  |  |  |  |

| **Self-management behaviors** | **≥ 5 times/week or more (4)** | **3-4 times / week (3)** | **1-2**  **times/ week (2)** | **Never practiced**  **(1)** |
| --- | --- | --- | --- | --- |
| **In terms of exercise** |  |  |  |  |
| 13. There is physical movement or activity in housework or occupation, such as housework/ gardening/farming/rice farming or walking to work until feeling moderately tired for at least 30 minutes at a time. |  |  |  |  |
| 14. Physical activities or recreational activities such as traditional sports, circle dancing, or rhythmic activities |  |  |  |  |
| 15. Observe any abnormal symptoms of the body while exercising, such as an abnormally fast heartbeat, chest tightness, shortness of breath, dizziness, faintness. |  |  |  |  |
| **In terms of stress management** |  |  |  |  |
| 16. Sleep 7-9 hours per day. |  |  |  |  |
| 17. Use methods of praying, meditating, making merit and worshiping Buddha. |  |  |  |  |
| 18. Use the method of consulting and talking with family members, talking with relatives or friends. |  |  |  |  |
| 19. Use the method of buying sleeping pills, sleeping vitamins, or drugs related to the nervous system to eat. |  |  |  |  |
| 20. Use self-control techniques, manage your emotions by practicing breathing exercises when you feel angry, or do activities with your family, such as eating, watching TV, or traveling. |  |  |  |  |
| **In terms of taking medication** |  |  |  |  |
| 21. Read the label on the front of the package before taking any medicine. |  |  |  |  |
| 22. Take medication according to the dosage and amount as per the treatment plan. |  |  |  |  |
| 23. When I forget to take my medicine, I take it as soon as I remember and double the dose at the next dose. |  |  |  |  |
| 24. In the past month, you have not taken your medication at the correct time (e.g. more than 1 hour before or after your usual dose). |  |  |  |  |
| 25. If there is any abnormality from taking medicine, I will immediately see a doctor. |  |  |  |  |

**Part 5: Elderly Quality of Life Questionnaire.**

Note: This questionnaire aims to assess the quality of life of the elderly in the past 1 month regarding opinions and experiences on various stories specified in each question that apply to you. You can put a check mark (/) in the space that applies to your opinion only one time.

| **Definition** |  |  |
| --- | --- | --- |
| (5) | Meaning | Most/Regularly |
| (4) | Meaning | Much |
| (3) | Meaning | Moderate |
| (2) | Meaning | Little |
| (1) | Meaning | Never |

| **Items** | **In the past month** | **1** | **2** | **3** | **4** | **5** |
| --- | --- | --- | --- | --- | --- | --- |
| 1. | Impairments to senses affect daily life  (How does the decline or loss of sensory/sensory function (e.g. hearing, vision, smell, taste, touch) affect your quality of life?) |  |  |  |  |  |
| 2. | Loss of sensory abilities affect participation in activities  (The decline or loss of function of a sensory/sensory system, such as hearing, vision, smell, taste, or touch, affects the ability to function, want, or participate in community activities.) |  |  |  |  |  |
| 3. | Freedom to make own decisions  (To what extent do you have the freedom to make your own decisions, such as deciding where to go or what activities to do?) |  |  |  |  |  |
| 4. | Feel in control of your future  (How much control and planning do you feel about your future?) |  |  |  |  |  |
| 5. | People around you are respectful of your freedom  (To what extent do you feel that the people around you respect your decisions, your independence of thought and decision-making?) |  |  |  |  |  |
| 6. | Concerned about the way you will die  (How concerned are you about your dying moments?) |  |  |  |  |  |
| 7. | Afraid of not being able to control death  (How worried are you about not being able to control your own death?) |  |  |  |  |  |
| 8. | Scared of dying  (How afraid are you of the possibility of death?) |  |  |  |  |  |
| 9. | Fear pain before death  (How much do you fear pain before death?) |  |  |  |  |  |

| **Items** | **In the past month** | **1** | **2** | **3** | **4** | **5** |
| --- | --- | --- | --- | --- | --- | --- |
| 10. | Problems with sensory functioning affect ability to interact  (How much do sensory/tactile systems (e.g., decreased hearing, blurred vision, abnormal smell, altered taste, abnormal touch) affect your interactions with others?) |  |  |  |  |  |
| 11. | Able to do things you’d like  (To what extent can you do or participate in activities that interest you?) |  |  |  |  |  |
| 12. | Satisfied with opportunities to continue achieving  (How satisfied are you with the opportunities you have received that will lead to success in your life?) |  |  |  |  |  |
| 13. | Received the recognition you deserve in life  (To what extent do you feel you deserve to have your life valued and respected?) |  |  |  |  |  |
| 14. | Have enough to do each day  (To what extent do you feel that you have enough to live your life?) |  |  |  |  |  |
| 15. | Satisfied with what you’ve achieved in life  (How satisfied are you with your success in life?) |  |  |  |  |  |
| 16. | Satisfied with the way you use your time  (How satisfied are you with your lifestyle?) |  |  |  |  |  |
| 17. | Satisfied with level of activity  (How satisfied are you with your ability to participate in activities with others?) |  |  |  |  |  |
| 18. | Satisfied with opportunity to participate in community  (How satisfied are you with the opportunities you have to participate in various activities in your community?) |  |  |  |  |  |
| 19. | Happy with things to look forward to  (How happy are you with the things you look forward to in the future, such as your children graduating or getting married?) |  |  |  |  |  |
| 20. | Rate sensory functioning  (How good do you think you are at your sensory/perceptual functioning (e.g. hearing, seeing, smelling, tasting, touching)? |  |  |  |  |  |
| 21. | Feel a sense of companionship in life  (To what extent do you feel you have friends (and are not alone)? |  |  |  |  |  |
| 22. | Experience love in your life  (In your life, how many experiences of love have you had?) |  |  |  |  |  |
| 23. | Opportunities to love  (In your life, how many opportunities have you had to love other people or things?) |  |  |  |  |  |
| 24. | Opportunities to be loved  (How much love do you have from your children?) |  |  |  |  |  |
